# Experimental Bracket Design Performance on Bonding and Polymerization of Orthodontic Composite

**DOI:** 10.1155/2024/7457900

**Published:** 2024-06-06

**Authors:** Alexandre Daher Yunes Salgado, Victor Pinheiro Feitosa, Manuela Timbó Farrapo, Tainah Oliveira Rifane, Lara Leal Ribeiro, Andressa Silva de Oliveira, Diego Lomonaco

**Affiliations:** ^1^ Dental School PPGO-UFC Federal University of Ceara, Fortaleza, Brazil; ^2^ Department of Operative Dentistry University of Iowa College of Dentistry, Iowa City, USA; ^3^ Department of Orthodontics Paulo Picanço School of Dentistry, Fortaleza, Brazil; ^4^ Department of Restorative Dentistry Piracicaba Dental School University of Campinas, Piracicaba, Brazil; ^5^ Department of Organic and Inorganic Chemistry Federal University of Ceara, Fortaleza, Brazil

## Abstract

**Objective:**

To evaluate the enamel bonding ability and orthodontic adhesive resin degree of conversion using the experimental bracket design. *Material and Methods*. Thirteen bovine teeth were used in the study. The experimental bracket was modified with a translucent region in the center of its body. After enamel etching, Orthocem orthodontic adhesive (FGM, Joinville, Brazil) was applied on the bracket base for bonding. The groups were divided as follows (*n* = 10 per group): (1) control (CB) with standard brackets and (2) spot bracket (SB) with experimental brackets featuring a 0.8 mm translucent region at the center using carbide bur. Shear bond strength (SBS) was evaluated after 24 hours in a universal testing machine and adhesive remnant index (ARI). The degree of conversion (DC) was analyzed using Raman spectroscopy (*n* = 3 per group). Data were then analyzed using Student's *t*-test and Mann–Whitney statistical methods.

**Results:**

The SB group exhibited a higher mean SBS (10.33 MPa) compared to the CB Group (8.77 MPa). However, there was no statistical difference between the groups (*p* = 0.376). Both SB and CB groups had a mean ARI score of 1. Raman analysis revealed a higher degree of conversion in the SB group (49.3%) compared to the CB group (25.9%).

**Conclusions:**

The experimental support showed a higher degree of adhesive conversion, although there was no significant increase in bond strength.

## 1. Introduction

The success of orthodontic treatment with fixed appliances is directly correlated with the ability of orthodontic accessories to securely adhere to the tooth structure [1–4]. This bonding process relies on the proper preparation of enamel with acid etching, aluminum oxide blasting, and primers, as well as adequate polymerization of the cured adhesive system and composite resins [1–4]. However, orthodontic accessory failure is documented in the literature, with prevalence ranging from 3.5% to 10% [1]. This issue is responsible for causing delays in treatment progress, patient discomfort, and dissatisfaction [2–4].

Literature suggests that an average shear bond strength (SBS) of 6 to 8 MPa is the minimum acceptable bonding strength for metallic brackets [1, 3]. Bonding has been achieved due to the evolution of orthodontic adhesive devices in recent decades, which has significantly contributed to a notable reduction in failure rates in the field of orthodontics, largely attributed to advances in adhesive systems and composite resins. Another factor has been the modification of the geometry of orthodontic attachment bases to improve mechanical retention between the adhesive agent and the device base [2–4].

Despite advances in restorative materials, the study of enamel adhesion remains important because metallic brackets pose a challenge due to their opaque nature, which limits the amount of light reaching the adhesive agent at the bracket base [2–4]. In the study by Sena et al. [5], it was demonstrated that the degree of conversion of orthodontic adhesive, located in the central region of the metallic bracket, falls below the ideal range, resulting in weaker bonding of this accessory due to lower light incidence during polymerization. Thus, current research is focused on accelerating polymerization and reducing clinical time [6–9], improving effectiveness [8, 9], and understanding how oral environmental factors may influence polymerization [10, 11].

Thus, an alternative to mitigate these consequences and increase monomer conversion in the central region of metal brackets is to modify orthodontic metal brackets with a central hole. This modification would allow direct exposure to light in this region, reducing the aforementioned factors. However, to date, no study has evaluated the correlation between bracket modification and its benefits in terms of enamel bond strength and degree of conversion.

Therefore, the study is aimed at developing and *in vitro* assessing an experimental metallic orthodontic bracket (spot bracket) modified in its central region to allow the passage of light from the light-curing device. The null hypothesis for the study is that no difference on enamel shear strength and degree of conversion between the control bracket and the spot bracket would be found.

## 2. Methods

### 2.1. Study Type

**Figure 1:**
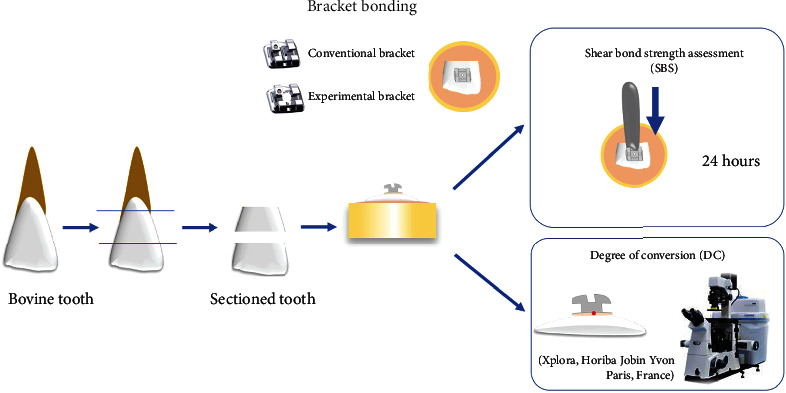
Illustration of the study design with sections of bovine teeth, enamel bonding procedure for brackets subjected to shear bond strength, and degree of conversion.

This randomized controlled *in vitro* study is aimed at comparing the shear bond strength, degree of conversion, and the adhesive remnant index (ARI) of orthodontic brackets. Two types of brackets were tested: conventional metallic brackets and modified brackets, with the latter referred to as “Spot Bracket.” The experimental design is illustrated in Figure 1.

### 2.2. Sample Preparation

To ensure uniformity and reduce interoperator variability, all sample preparation procedures were conducted by a single operator. This approach was adopted to enhance the reliability and reproducibility of the experimental results. Thirteen bovine teeth were obtained from a slaughterhouse and then were prepared by cutting their roots at the cemento-enamel junction using Cut Master (Londrina, Brazil). The buccal surface is exposed. The samples were embedded with acrylic resin (JET, São Paulo, Brazil) in polyvinyl chloride (PVC) rings with only the vestibular surface exposed.

### 2.3. Bonding Procedure

**Figure 2:**
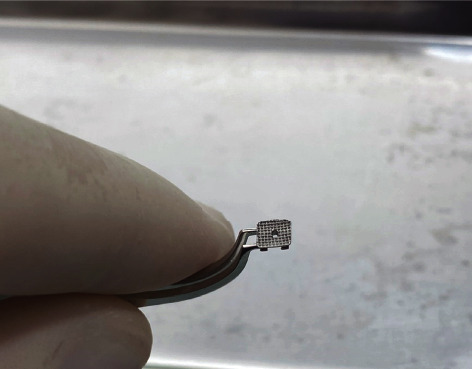
Experimental bracket with light hole.

**Figure 3:**
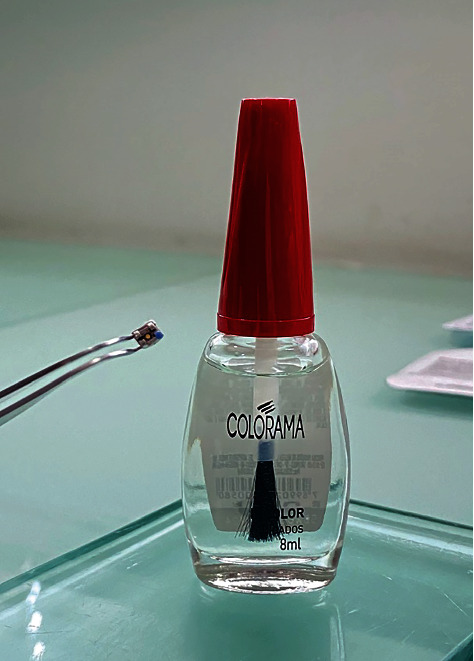
Experimental bracket light hole covered with clear nail polish.

**Figure 4:**
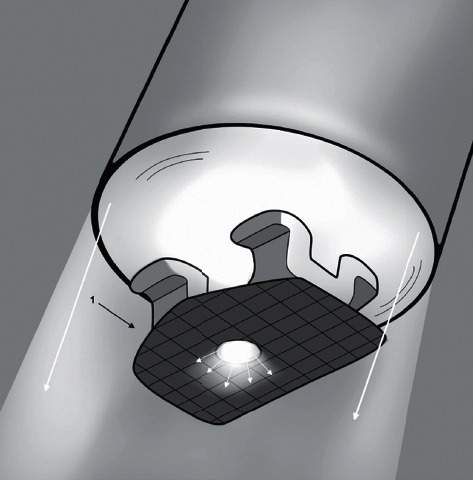
Schematic representation of the light passage through the experimental bracket.

The dental prophylaxis was conducted using pumice and a Robson brush for 30 seconds, followed by rinsing for 60 seconds. The buccal surfaces of the bovine teeth were etched using 37% phosphoric acid gel (Condac 37, FGM, Joinville, Brazil) for 30 seconds. Subsequently, the teeth were rinsed for 60 seconds and dried. Roth Light prescription upper central incisor brackets (Morelli, Sorocaba, São Paulo, Brazil) were divided into two groups (*n* = 10 per group): control group (CB) and spot bracket group (SB). Control group brackets were bonded without any modification to their structure. For the SB group, a hole was manually created in the central portion of the bracket using a 0.8 mm diameter round carbide bur (FG 1, GDK Densell Dental, Buenos Aires, Argentina), as shown in Figures 2 and 3. To avoid any adhesive leakage or overflow through the hole during placement, a thin layer of clear nail polish (Colorama Esmaltes, São Paulo, Brazil) was applied to cover and seal the hole while still permitting the passage of light (Figure 4). The base of the brackets had an area of 15.08 mm^2^. Regarding the experimental bracket with the 0.8 mm diameter hole, the device base area was 13.08 mm^2^ (excluding the hole).

Orthocem orthodontic adhesive (FGM, Joinville, Brazil) (Table 1) was used for bonding. The adhesive was directly applied to the base of the brackets, and they were positioned on the buccal surface of the bovine teeth. Utilizing the back of the bracket holder (Morelli, Sorocaba, São Paulo, Brazil), the brackets were pressed against the teeth to allow excess resin to be removed using a clinical probe. The adhesive was then cured with a Valo unit (Ultradent, South Jordan, USA) for 20 seconds, positioned as close as possible and perpendicular to the buccal surface of the bracket. Subsequently, the samples were stored in distilled water at 37°C for 24 hours for the subsequent shear bond strength (SBS) test.

### 2.4. Shear Bond Strength Assessment (SBS) and Analysis of the Adhesive Remnant Index (ARI)

The samples were then subjected to SBS tests (*n* = 10 per group) in a universal testing machine (4411; Instron Corp, São José dos Pinhais, Paraná, Brazil) with 0.5 mm/min speed, according to the methodology proposed by Borges et al. [12]. The sample size was calculated using the same number of specimens per group according to previous studies, and ten teeth were cut in half totaling twenty samples, *n* = 10 for each group [11, 12]. The active tip of the chisel was positioned in an occlusogingival direction in contact with the bracket, between the tie-wing and the base, close to the base. The breaking loads were measured in newtons (N) and then converted into megapascals (MPaN/mm^2^). Based on the bracket base dimensions, the bonding area was calculated as 11.34 mm^2^.

After the SBS tests, each sample was analyzed on the surface of the dental enamel with a stereoscopic magnifying glass (20X-80X, OMAX, Buenos Aires, Argentina) at 25x magnification to determine the adhesive remnant index in the enamel: score (1)—no adhesive remained on the enamel; score 2—less than 50% of the adhesive remained on enamel; score (3)—more than 50% of the adhesive remained on enamel; score (4)—all adhesives remained on the enamel. The ARI was assessed by a single operator and calibrated in relation to the area of the bracket and the remaining adhesive material. Descriptive analysis was used to report the failure modes with their respective percentages.

### 2.5. Degree of Conversion (DC)

**Figure 5:**
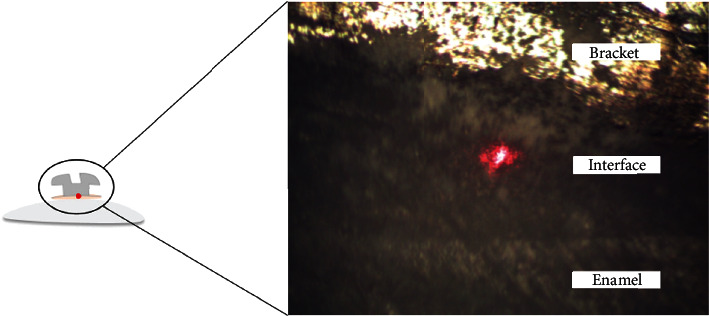
Raman analysis image of the enamel and bracket bonding interface.

According to the methodology proposed by Araújo-Neto et al. [13], subsequently, after 24 hours of storage in distilled water at 37°C, three additional samples (not submitted in the SBS test) from each group (*n* = 3) were sectioned in the cross-section to expose the bracket-enamel interface (Figure 5). This *in situ* analysis of the degree of conversion of the specimens was performed by micro-Raman spectroscopy (Xplora, Horiba Jobin Yvon, France), with a HeNe laser with power set to 3.2 mW and a wavelength of 532 nm, in 10 seconds of acquisition time. It was employed with 1.5 *μ*m spatial resolution and 2.5 cm^−1^ spectral resolution associated with a 10x magnification lens (Olympus, London, UK). Firstly, it collected the spectra of unpolymerized resin (three points), and subsequently, the spectra of the polymerized resin were obtained at three different sites along the bracket-enamel interface. The calculation performed to observe the degree of conversion is based on the following equation:

(1) $$\begin{array}{c} \text{DC}\%=\left(1-\frac{R\text{ cured}}{R\text{ uncured}}\right)\times 100. \end{array}$$

The “R” is the ratio between the heights of 1637 cm^−1^ (C=C) and 1610 cm^−1^ (aromatic) peaks of uncured and light-cured material.

### 2.6. Statistical Analysis

The sample size for each test had a minimal power of 0.8 at a significance level of 5% (*β* = 0.2). Data and sample size calculation were analyzed using SPSS software, version 17 (SPSS Inc., Chicago, IL). SBS results were previously tested for normality (Shapiro-Wilk analysis) and homoscedasticity (for variance analysis). After the normality test, the data were statistically analyzed using Student's *t*-test analysis with a significance level of 5%.

## 3. Results

### 3.1. Shear Bond Strength Assessment (SBS) and Adhesive Remnant Index Analysis (ARI)

**Figure 6:**
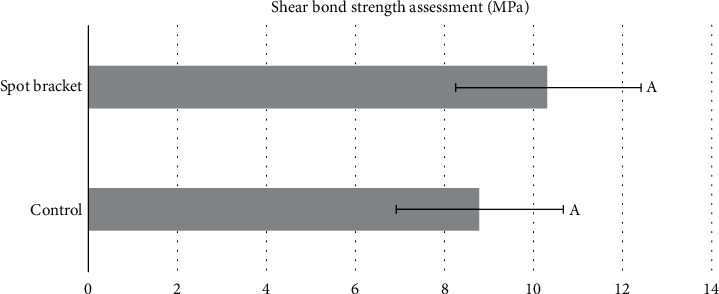
Mean and standard deviation of shear strength (SBS) in MPa with no statistical difference between groups (*p* = 0.376).

**Figure 7:**
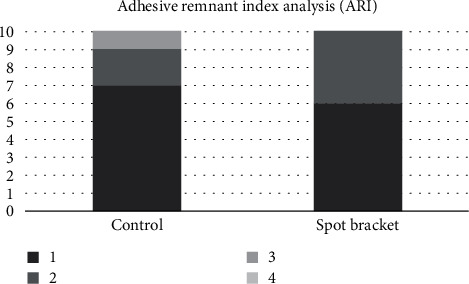
Distribution of the adhesive remaining index (ARI) scores after shear bond strength.

The results of the shear bond strength (SBS) tests are presented in Figure 6. The control group (CB) exhibited a lower SBS mean of 8.77 MPa, while the spot bracket group (SB) showed a higher SBS mean of 10.33 MPa (Figure 6). However, there was no statistical difference between the groups (*p* = 0.376). The adhesive remnant index (ARI) results are summarized in Figure 7. Both the CB and SB groups predominantly received ARI scores of 1, indicating the absence of adhesive layer residue on the enamel surface.

### 3.2. Degree of Conversion (DC)

**Figure 8:**
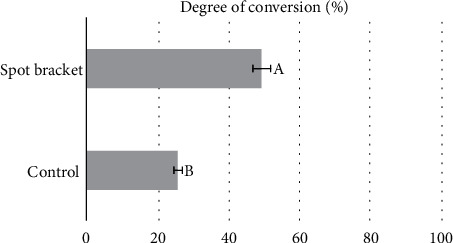
Degree of conversion (DC) results with a statistically significant difference (*p* = 0.002).

The degree of conversion (DC) results are summarized in Figure 8. The Raman spectroscopy analysis revealed a significantly higher degree of conversion in the spot bracket group (SB) reaching 49.31%, compared to the control group (CB), which scored 25.90%. This difference was statistically significant (*p* = 0.002).

## 4. Discussion

Based on the findings of the study, the modification of the bracket (spot bracket) yielded a higher degree of conversion without exhibiting any disparity in shear bond strength (SBS) to enamel, with values ranging from 8 to 10 MPa. Thus, the null hypothesis was negated. Sena et al.'s investigation [5] delineated that the orthodontic resin situated in the central region of the bracket base manifests a diminished degree of conversion in comparison to the margins postphotoactivation. This phenomenon may precipitate a weakened adhesion region at the bracket's base, potentially culminating in failures in orthodontic device fixation [14–17]. Consequently, various methodologies, strategies, and alternatives are being explored to augment bonding efficacy and the degree of conversion.

The assessed brackets evinced no noteworthy variance in SBS values at the 24-hour mark. This observation could be attributed to the experimental configuration of the bracket, which may entail a diminished mesh contact area at the base, conceivably impinging upon SBS and warranting the congruent outcomes. Additionally, the perforation engendered may precipitate a reduction in device stability and structural rigidity, thereby engendering bracket spatial geometry distortion that could potentially impede precision in orthodontic manipulation. Furthermore, the utilization of enamel as an uneven filling may engender superfluous material accumulation on the bracket mesh, obstructing enamel adhesion. In the adhesive remnant index (ARI) scrutiny (Figure 6), both cohorts evinced a preponderance of score 1, indicative of adhesive-enamel interface failure. Such an outcome may confer clinical advantages by mitigating the risk of healthy enamel removal during bracket detachment.

In view of this, the use of light emitting diode (LED) in high irradiance mode has been advocated in orthodontic treatment to reduce the photoactivation time as it can reach up to 3,000 mW/cm^2^ in periods of 3 to 5 seconds [4, 16–18]. However, research findings are contradictory, with most studies suggesting that this reduction in time and high power may adversely affect the adhesive bond to enamel and its physicochemical properties [18–21]. Moreover, prolonged exposure may pose risks such as tissue burns, soft tissue irritation, and an increase in dental pulp temperature [15, 17, 18].

The inferior performance of orthodontic resin in this photoactivation mode is related to the photoinitiator present in its composition and its reactivity. In most orthodontic resins, the photoinitiator used is camphorquinone (CQ), classified as a Norrish II type photoinitiator, requiring an efficient hydrogen donor to enhance its efficiency with a maximum absorption peak at 470 nm [21, 22], being sensitive to blue light. The CQ/amine reaction is slow in a short period of time and high power, resulting in incomplete conversion of resin monomers. Therefore, since light-curing units with a light intensity of 1000-1500 mW/cm^2^ are capable of producing sufficient light intensity for bonding, the use of devices in more potent modes is not necessary and does not seem to be as appropriate.

Another crucial factor to consider is the correlation between the type of bracket and its ability to transmit and/or reflect light. Even in the case of aesthetic brackets, such as polycarbonate, polycrystalline, and monocrystalline brackets, greater translucency may be observed. However, there is still a significant reduction in luminosity when photoactivation is performed exclusively on the bracket surface, which may lead to unsatisfactory bonding results [20, 21, 23, 24]. In the case of metal brackets, their opacity prevents the passage of light. These factors may result in a decrease in the radiant emission values required to activate the photoinitiators.

To address the aforementioned challenge, various photoactivation techniques are recommended, including transillumination and positioning the light-curing unit on the mesial, distal, and incisal surfaces [6, 20, 21, 25]. In an attempt to replicate challenging clinical scenarios, where optimal positioning of the light-curing unit is unlikely, such as when bonding the buccal surface of maxillary second molars, this study adopted a perpendicular positioning of the unit in relation to the buccal surface of the bracket. Additionally, some light-curing devices, such as the Valo, feature large-area light tips (8 mm in this specific model), capable of covering the entire body of the bracket and its surroundings. This feature enables the light to reach all sides of the bracket, even when not directed directly.

In addition to bracket geometry and composition, other factors also influence photopolymerization, with tooth structure being a crucial aspect to consider. Dental enamel has a high light transmittance capacity due to its high refractive index [23]. It consists of prisms and interprisms arranged in parallel [23, 25], which can facilitate light diffusion [26], as well as interprismatic spaces. This study was conducted using bovine teeth due to the similarity of enamel microstructure to that of human teeth [27, 28]. Several studies [28–31] have found no significant differences in luminescence and refractive capacity between bovine and human enamel, demonstrating the feasibility of conducting studies on the adhesion of orthodontic devices to dental structures.

The organization of enamel prisms may result in random light dispersion along its surface rather than concentrated reflection, potentially favoring adhesion [28–30]. This behavior of light may explain why standard brackets showed similar SBS results to the experimental ones. Even when photopolymerization is performed perpendicularly to the bracket body, blocking most of the light, the surrounding light that reaches the enamel surface is refracted and reaches the adhesive at the base. Another relevant aspect that may have contributed to the similarity in results between the groups is the fact that orthodontic adhesives typically contain a lower percentage of filler compared to normal composites, which increases light propagation and favors photopolymerization. Additionally, other variables influence the bond strength of orthodontic materials, such as curing light output [32–35]. Although no statistical difference in shear bond strength was observed between the experimental and conventional brackets, clinically, this higher adhesive conversion rate may translate into more efficient bonding, less prone to failures, thereby favoring a more predictable treatment with fewer complications [1].

The limitations of the study included the evaluation being restricted to a 24-hour period, the small sample size, and the absence of other types of brackets for comparison. Although the initial results appear promising, future studies are crucial to confirm whether the experimental spot bracket indeed offers superior performance. The experimental design of the bracket may present a reduced mesh contact area in the base, which can negatively influence bond strength. Additionally, the hole created through the bracket body may result in diminished stability and structural rigidity of the device, leading to distortion of the bracket's spatial geometry that could potentially alter its precision in orthodontic movement. Furthermore, the use of nail polish as a filler for the hole was a temporary measure to enable *in vitro* testing of the hypothesis. However, nail polish is unsuitable for prospective clinical evaluations, necessitating a new translucent and biocompatible material for filling the inner orifice.

Hence, the most significant finding of this experimental bracket design (spot bracket) could be regarded as the potential for a higher rate of conversion of orthodontic adhesive. The brackets utilized in this study were intended for upper central incisors. However, considering brackets with larger dimensions, such as those designed for molars, the difference in monomer conversion rate could potentially be even more significant. This is due to the larger opaque area of the bracket extending to the center of its base.

## 5. Conclusion

The experimental brackets (spot bracket) demonstrated a higher degree of adhesive conversion. However, there was no statistically significant improvement in bond strength to enamel.

## Figures and Tables

**Table 1:** Orthocem manufacturer, composition, and batch number.

Material	Manufacturer	Composition	Batch number
Orthocem orthodontic adhesive	FGM	Silane-treated silicon dioxide, bisphenol A diglycidyl ether methacrylate (BisGMA), triethylene glycol dimethacrylate (TEGDMA), methacrylated phosphate monomer, sodium fluorite, and camphorquinone	260821

## Data Availability

Research data can be presented upon a reasonable request.
